# In Vitro Comparative Evaluation of Different Types of Impression Trays and Impression Materials on the Accuracy of Open Tray Implant Impressions: A Pilot Study

**DOI:** 10.1155/2017/6306530

**Published:** 2017-02-27

**Authors:** Sonam Gupta, Aparna Ichalangod Narayan, Dhanasekar Balakrishnan

**Affiliations:** Department of Prosthodontics and Crown & Bridge, Manipal College of Dental Sciences, Manipal University, Manipal, Karnataka, India

## Abstract

*Purpose*. For a precise fit of multiple implant framework, having an accurate definitive cast is imperative. The present study evaluated dimensional accuracy of master casts obtained using different impression trays and materials with open tray impression technique.* Materials and Methods*. A machined aluminum reference model with four parallel implant analogues was fabricated. Forty implant level impressions were made. Eight groups (*n* = 5) were tested using impression materials (polyether and vinylsiloxanether) and four types of impression trays, two being custom (self-cure acrylic and light cure acrylic) and two being stock (plastic and metal). The interimplant distances were measured on master casts using a coordinate measuring machine. The collected data was compared with a standard reference model and was statistically analyzed using two-way ANOVA.* Results*. Statistically significant difference (*p* < 0.05) was found between the two impression materials. However, the difference seen was small (36 *μ*m) irrespective of the tray type used. No significant difference (*p* > 0.05) was observed between varied stock and custom trays.* Conclusions*. The polyether impression material proved to be more accurate than vinylsiloxanether impression material. The rigid nonperforated stock trays, both plastic and metal, could be an alternative for custom trays for multi-implant impressions when used with medium viscosity impression materials.

## 1. Introduction

Oral rehabilitation of partial and complete edentulism with osseointegrated implants has presently become a conventional treatment and its longitudinal effectiveness as one of the viable treatment modalities has been proven by several clinical studies [1, 2]. Nevertheless, the advent of implant dentistry has exemplified the urgency for a precisely fitting final prosthesis [3].

Since endosseous implants are functionally ankylosed, they are in direct contact with the bone, as a result lack inherent mobility of the periodontal ligament [4]. On the contrary natural teeth have the ability to resist horizontal, vertical, and rotational forces because of the stress bearing capacity of the periodontal ligament. Consequently, misfit of these frameworks could load the implants unnecessarily and jeopardize their longevity. Hence, precise fit of the framework becomes more critical when final restorations are attached to implants as compared to natural teeth [5]. Any misalignment between the osseointegrated implants and its superstructure may invoke stresses in the dentures, implants, and the peri-implant bone matrix [6, 7].

For a precise fit of the implant framework, having an accurate definitive cast is imperative [8–12], which further depends on wide range of variables, the dental impression recorded, the physical and mechanical properties of impression material [13], the impression technique employed [14], the selection of the impression tray [15], the die material's accuracy, machining forbearance of prosthetic components [16], and the depth [17] and angulation [18] of implant placement [8]. Nevertheless, the foremost objective of an implant impression, particularly involving multiple implants, is to transfer and replicate the intraoral position of implant analogues to the other associated structures existing as precisely as possible [19].

Majority of impression materials when handled appropriately are primed of yielding clinically satisfactory impressions [20, 21]. One considerate aspect that has not yet been researched in detail is the proper selection of the impression trays for implant impressions for completely edentulous situations [22–27]. The accuracy of the resultant impressions, however, is contingent to the combination of the impression material and tray used. Moreover, the deformed trays may lead to distortion of impressions, which seems to be acceptable on visual examination and is found deficient only during insertion of the respective prosthesis [28].

Although a number of impression materials are manufactured with a variety of different consistencies, comprehensive evaluation is necessary to document the rigidity and accuracy of these materials, particularly those employed for direct implant impression technique. Many published studies have validated usage of polyether as an impression material for multiunit implant-retained restorations in completely edentulous situations for its properties of low strain during compression with an optimum Shore A hardness [14, 29–38]. In contrast, use of addition silicone as an impression material permits easy removal once the impression is set due to its more favorable modulus of elasticity and therefore has also been recommended as a preferred material for implant impressions using direct technique [13, 39, 40]. Henceforth, it could be concluded that polyether and addition silicone are the most commonly recommended materials of choice for multi-implant impressions.

Recently, advances in elastomeric chemistries have given origin to a new generation of impression material that is a combination of polyvinylsiloxane and polyether material called vinylsiloxanether, which has been made available commercially. It combines some of the most desired properties of both into one material. This has been claimed by the manufacturer to possess acceptable mechanical and flow properties, besides its unique wetting characteristics. Moreover, the accuracy of impressions is improved by its enhanced hydrophilicity resulting in improved flow with recording of finer details of impression [41]. However, there is an insufficient scientific evidence to prove its clinical efficiency as an impression material for multiunit implant impressions. Therefore, the aim of this in vitro study was to evaluate and compare the linear and cross-arch dimensional accuracy of casts obtained using different impression trays and impression materials on the accuracy of open tray implant level impressions using splinted transfer copings. The two null hypotheses tested were as follows.

1. The first null hypothesis was that there would be no significant difference in the dimensional accuracy of casts obtained in terms of linear and cross-arch discrepancies between polyether and vinylsiloxanether impression material using open tray impression technique with splinted transfer copings.
2. The second null hypothesis tested was that there would be no significant difference in the dimensional accuracy of casts obtained in terms of linear and cross-arch discrepancies between varied stock and custom trays used for open tray impression technique with splinted transfer copings.

## 2. Materials and Methods

### 2.1. Study Design

The Institutional Ethical Committee approval for the study (ECR/146/Inst/KA/2013) was attained. The study compared and evaluated two different types of impression materials: PE (3M ESPE, Soft Monophase) and VSXE (Identium Medium, Kettenbach GmbH). Groups using these two impression materials were further subdivided into eight experimental subgroups relating four types of different impression trays: self-cure custom tray (Rapid Repair, Dentsply,), light cured custom tray (Individuo Lux), plastic stock tray (O-Tray disposable nonperforated, Dentaurum), and metal stock tray (CAT SS edentulous nonperforated, Liberaltraders).

For each group, five implant level impressions of the reference model were made, thus comprising twenty impressions for each impression material and congruently comprising twenty impressions for each stock and custom trays in total making of forty impressions. Standardized experimental casts were made for each impression. All impressions were made with open tray impression technique using splinted transfer copings. The measurements of linear and cross-arch distances of the experimental casts were obtained and further the discrepancies were deliberated in relation to the reference model. The distortion values for varied impression trays and materials were equated using statistical analysis.

### 2.2. Fabrication of the Reference Model

**Figure 1:**
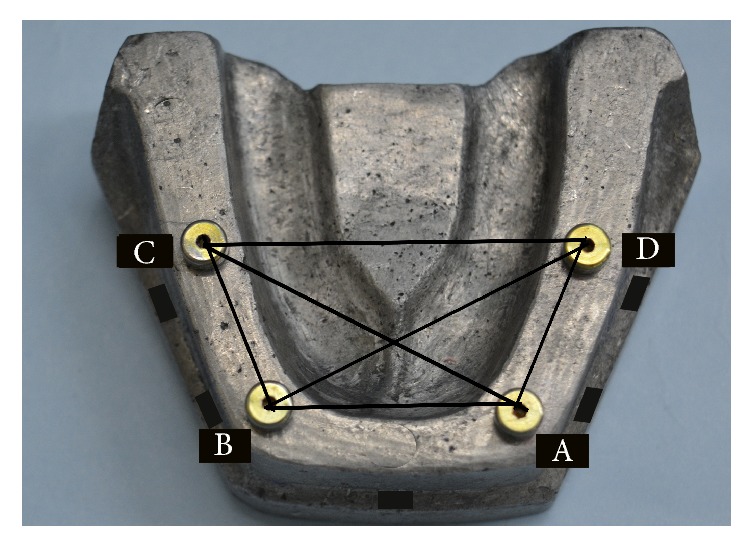
Reference model with implants and healing abutments in place.

A machined aluminum reference model was fabricated to simulate the mandibular edentulous arch to serve as the master cast. Four parallel holes of equal size were drilled perpendicular to the arch in the canine and first molar region on both sides. Next, implant replicas (NobelReplace, RP, 4.3 × 13 mm) were placed parallel to each other in the drilled holes. The healing abutments were hand tightened upon the implants with the help of UniGrip (NobelReplace) screwdriver for the subsequent measurements (Figure 1).

### 2.3. Custom Tray Fabrication

#### 2.3.1. Self-Cure Custom Tray

Five stops, one anterior and two posterior on either side of arch, were prepared in the vestibular region of the reference model to ensure correct orientation of custom impression trays. Wax spacer of 5 mm thickness was adapted uniformly upon the implant reference model [42–46]. To ensure uniform thickness of spacer for all custom trays, the implant reference model along with wax spacer was duplicated using putty impression material and poured in die stone. The duplicated cast was used for the fabrication of the trays. The self-cure acrylic was mixed according to manufacturer's instructions and was secured to the duplicated cast with a uniform thickness of 2 mm. Further, the trays were placed in pressure pot for 30 minutes to minimize the porosities and to obtain better adaptation.

#### 2.3.2. Light Cure Custom Tray

As stated above, the light cure trays were fabricated in a similar manner. The light cure sheet was adapted to the cast. Following the manufacturer's instructions, the tray was kept in the light cure-curing unit for 5 minutes with the tray positioned on the cast. Further, after initial curing, the tray was removed from the cast and then the impression surface of the tray was cured for 5 minutes.

For each sample, individual self-cure and light cure custom trays were made. All the trays were fabricated 24 hours prior to their use [47–50]. Four windows were drilled in the trays corresponding to the implant sites to allow access to the coping screws for the open tray impression technique.

### 2.4. Stock Trays

The nonperforated plastic and metal trays used for completely edentulous impressions were obtained from the manufacturer. All sample impressions were made with single tray. Further, similar to custom trays, four windows were drilled in both the stock trays to allow access to the coping screws for the open tray impression technique.

### 2.5. Impression Making

All the open tray impressions for both the stock and custom trays were performed by the same experienced prosthodontist and certified implantologist. To make implant level impressions, tapered open tray impression copings (NobelReplace RP, 4.3 mm) were secured to the implant replicas and using the UniGrip screwdriver (28 mm long, NobelReplace), the impression coping screw was hand tightened.

**Figure 2:**
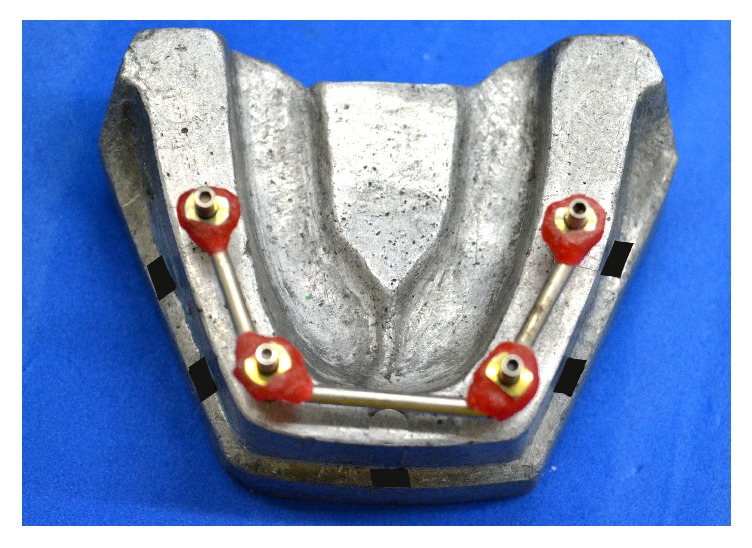
Metal splinted tapered impression copings.

All the four impression copings were splinted using an incremental application technique, wherein small amounts of autoploymerizing acrylic resin (Pattern Resin GC) were added to the shanks of 2 mm diameter burs and impression copings with brush bead method, until the square surfaces of the copings were fully covered (Figure 2) [51]. The openings in the tray were blocked with help of modeling wax to prevent outflow of impression material. Subsequent to the tray adhesive application on the tissue surface and circumference of the trays, impression material was mixed and dispensed using automatic mixing unit (Pentamix-2, 3M ESPE) into a metal syringe to record the finer details around the coping and further was loaded into the custom tray. Material was injected around the impression coping following which, the loaded impression tray was seated on the reference model with gentle finger pressure. Tray was held for 6 minutes on the reference model. Further, the tray was removed after the material was set completely and was examined for voids and defects or inadequacy around the copings.

### 2.6. Experimental Casts Fabrication

The same operator trained in implant laboratory procedures fabricated all the experimental casts. The implant replicas were secured to the tapered open tray impression copings, and coping screw was hand tightened using the UniGrip (NobelReplace) screwdriver. Care was taken to ensure the proper seating of the implant replicas in the impression. The impressions were boxed with the modeling wax to ensure the uniformity of the sample. 23 mL of water was used to mix 100 gms of type IV dental stone (Kalrock, Kalabhai, Karlson Pvt Ltd) as per manufacturer's instructions. The die stone was slowly scattered into the water in a plastic bowl and was hand spatulated for 10 seconds with a stainless steel spatula. Further, to obtain a creamy bubble-free uniform mix, the die stone was spatulated in mechanical vacuum mixer. The mixed die stone was judiciously poured along the margins of the impression to flow into the innermost segment with the impression positioned on the vibrator in a tilted manner. The die stone was added over the impression to fill it completely up till its periphery. After complete pouring of the impression, casts were left undisturbed to set for 30 min. The casts were then separated from the impression and were allowed to dry.

### 2.7. Measurement Protocol

All forty experimental casts were measured and examined for linear and cross-arch dimensional accuracy by means of a coordinate measuring machine (CMM) (Carl Zeiss, Contura G2) connected to computer. The CMM is capable of recording the *x*-, *y*-, and *z*-axis to an accuracy of 0.0018 mm (1.8 *μ*m) according to the manufacturer. Six measurements were obtained for every single cast, and the mean values were computed. The same operator performed all the measurements.

**Figure 3:**
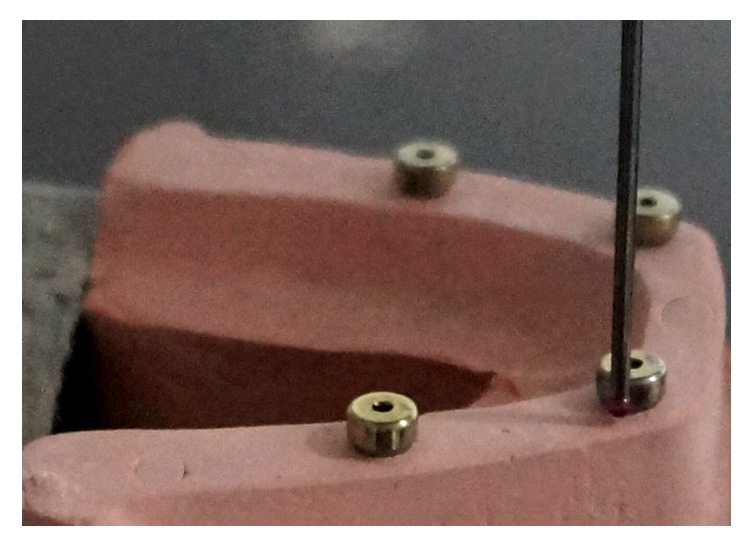
Measurement of coordinates of healing abutments using the CMM.

**Figure 4:**
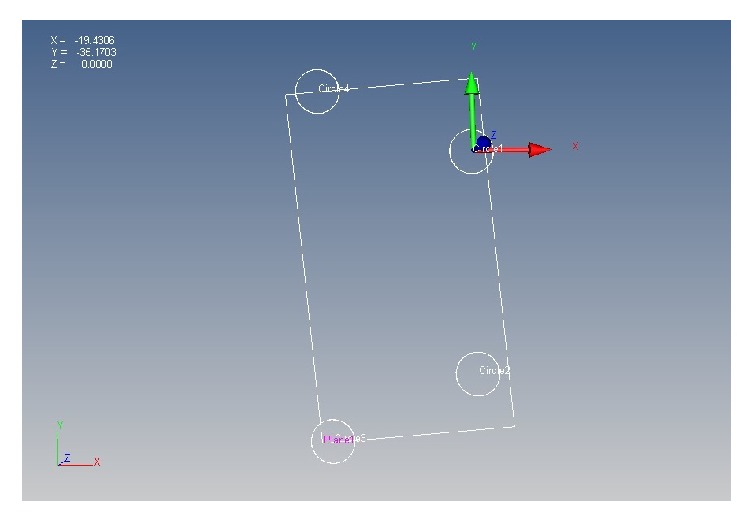
Establishment of coordinate system for measurements.

The healing abutments were secured to the implant replicas in the reference model and were hand tightened using UniGrip screw driver (NobelReplace) and were denoted sequentially A to D (from left to right). When locating the coordinates, the reference model and experimental casts were secured to the base. The centric of each abutment was then traced using a CMM probe with a diameter of 0.5 mm by touching numerous points on the perimeter of the external surface of the healing abutment (Figure 3). CMM software was used for geometric transfer and data handling. The centric of healing abutment A (Figure 4), which is present on the right side of the cast, was established as the starting point of the coordinated system (0,0, 0) for all the measurements. The *XY* plane was formed by the planar surface encompassing it. An imaginary *XZ* line was contemplated between the centers of the analogue A and D. The *XZ* plane was perpendicular to *XY* plane. Therefore, the center of analogue A was laid on the origin (0,0, 0) and the center of analogue D was laid on the *XZ* plane (*X*, 0, *Z*). For each analogue in the reference model as well as the definitive casts, CMM measured the Cartesian coordinates (*X*, *Y* and *Z*) of each analogue with respect to the determined reference axis. In order to define the Euclidean distance between each pair of analogues, the difference between their coordinate values in each dimension was deliberated. Using the Pythagorean theorem for a three-dimensional model, the six distance values [(*x*^2^ + *y*^2^ + *z*^2^)1/2] were measured (Figure 1), for the reference model and for every single experimental casts between the centric of healing abutments A and B, B and C, C and D, A and D, and A and C, and B and D. The mean average values obtained from the casts were related with the standard values attained from the reference model and the discrepancies were computed.

### 2.8. Statistical Analysis

The comparative values of the linear and cross-arch discrepancies were used to estimate the overall accuracy of the experimental casts in relation to the reference model. A parametric test was used to evaluate and compare the data statistically. The two-way analysis of variance was used to evaluate the influence of different impression materials and impression trays at a significance level of .05 (SPSS version 20, IBM). The mean descriptive values of different impression trays and impression materials were also obtained.

## 3. Results

**Figure 5:**
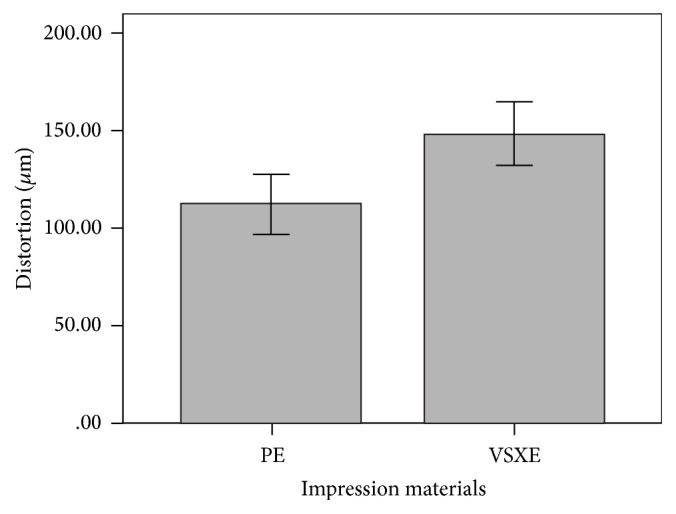
Descriptive mean analysis of test impression materials (*μ*m).

**Figure 6:**
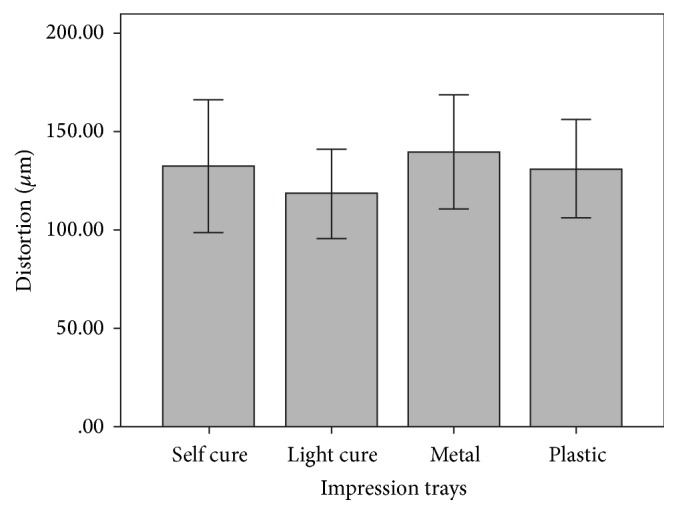
Descriptive mean analysis of test impression materials (*μ*m).

A parametric test, two-way analysis of variance, performed to validate the results is provided in Table 1. The mean descriptive values of distortion for both impression materials and impression trays were obtained, provided in Table 2 (Figure 5) and Table 3 (Figure 6), respectively. The linear and cross-arch discrepancies varied significantly for PE and VSXE impression material (*p* = 0.002). Statistically, between the different impression materials groups, significant differences were established (*p* < 0.05) without considering type of tray used as a factor, between PE and VSXE. The casts obtained from impressions made with PE impression material (112 *μ*m) proved to be more accurate than casts obtained from VSXE impression material (148 *μ*m). Mean discrepancy of 36 um was observed between the two materials. The two impression materials groups with similar stock and custom tray materials exhibited no significant differences amongst any of the combinations. However, no statistically significant difference was observed between different stock and custom trays (*p* > 0.05), irrespective of the impression material used. When the mean descriptive values were compared, the light cured tray displayed least distortion (118 *μ*m) among both custom and stock trays but was statistically insignificant. The stock trays, both polycarbonate and stainless steel, showed similar accuracy compared to the custom trays.

## 4. Discussion

With regard to the results obtained, the first null hypothesis was rejected that there would be no significant difference in the dimensional accuracy of cast in terms of linear and cross-arch discrepancies between PE and VSXE impression material. The casts obtained from PE proved to be more accurate when compared to the VSXE utilizing splinted open tray impression technique, with parallel implant placement. Enkling et al. [52] investigated and compared different impression materials using open tray implant impression technique, which included PE, VSXE with concurrent splinting of impression copings with A-silicone. The results of his study demonstrated that VSXE with respect to dentists, patients, and technician assessment ascertained to be similar or superior to the PE. Further, the results of the study conducted by Vojdani et al. [18] were in line with the study of Enkling et al. exhibiting no difference between PE and VSXE for multi-implant impressions with parallel implant placement. However, results of this in vitro study were contradictory to the above-mentioned studies. According to Del'acqua et al. [53], polyether should be the material of choice to achieve a more accurate orientation of implant analogues in laboratory master casts. The author also stated that the material rigidity prevents displacement of impression copings within the impression material.

Nevertheless, for the impression materials to yield clinically acceptable impressions [20, 21] proper selection of impression trays becomes critically important [15, 28]. Broadly, impression trays are classified as custom trays made specifically for the individual and stock trays that are commercially available in varied sizes. The stock trays can be metal and plastic trays [28]. Custom trays are fabricated from acrylic resin, which can be heat cured, cold cured, or visible light cured.

Many authors have reviewed the literature and therefore have achieved precise results with custom trays. This could be attributed to their properties of good adhesion with the impression material, dimensionally stable, allowing uniform thickness of the impression material and exhibiting adequate rigidity to resist distortion [25, 26, 54, 55].

However, plentiful number of stock trays is commercially available and they eliminate the need of making primary impression and subsequent primary casts and special trays thus saving a lot of chair-side time and patient discomfort [26, 56].

Burns et al. investigated the accuracy of stock and custom trays on open tray implant impressions and demonstrated better accuracy with custom trays as compared to stock [26]. Cho and Chee [28] in their study evaluated the stiffness and resistance to distortion of six disposable plastic stock trays and a metal stock tray. The authors advocated avoiding high viscosity impression material with disposable plastic stock trays.

In the present study, statistically similar results were obtained using different stock and custom trays. Therefore, the second null hypothesis was accepted as there would be no significant difference in the dimensional accuracy of casts obtained from varied stock and custom trays, and the decision of which impression tray to be used can be scrutinized by the clinician himself.

The results of the present study were contradictory to the above-mentioned studies and this could be comprehended by the usage of good quality metal (CAT SS edentulous nonperforated, Liberal Traders) and plastic (O-Tray disposable nonperforated, Dentaurum) plastic trays, which were appropriately chosen for this study based on the manner in which it conformed to the size of the reference model. It also exhibited sufficient rigidity to resist the deformation during use of polyether and polyvinylsiloxane impression material. The mean distortion values for both the test impressions made with plastic stock trays were 10 *μ*m lesser than the impressions made with metal trays. This could be attributed to the fact that the impression materials used for this study were of medium consistency, which allowed easier removal from the reference model as they offered less resistance to removal of set impression.

Del'acqua et al. [57] evaluated the rigidity of stock trays and compared plastic and metal trays on the accuracy of implant impressions. The author also stated that fabrication of custom trays are impractical in routine clinical settings because of the association of additional time and cost involved; therefore usage of stock trays warranting practicality is preferred by the clinician. He also inferred that stock metal trays exhibited more accuracy as compared to plastic especially when high viscosity impression material is used.

Among the tested custom trays, light cured trays displayed least distortion as compared to self-cure acrylic trays. Light cured custom trays also showed best results in terms of distortion when compared with stock trays. This could be attributed to the uniform thickness of the trays, which allowed uniform distribution of impression material. When compared with self-cure resin tray, the wastage of the material during fabrication of the tray was less and the associated polymerization shrinkage was reduced due to the uniform thickness of the light cure sheets. However, the difference was not statistically significant.

One reason to use the splinted tapered impression copings with metal burs in conjugation with autopolymerising resin was to reduce the probability of permanent displacement of copings. Further, the incremental addition of resin to the shanks of right-angle burs using bead brush technique could withstand the forces of distortion better that develops following the recovery of impression. Moreover, metal-splinted impression copings avoid the additional step of sectioning, rejoining, and associated polymerization shrinkage [58].

However, the distortion exhibited may not have any clinical significance, as it has been quoted in previous literature that a difference of up to 100–150 *μ*m is acceptable. The distortion values for both impression materials and impression trays were well within the range, which have been substantiated by numerous clinical studies. Therefore, it can be concluded that specific composition and viscoelastic properties of the impression materials play a crucial role in the impression accuracy. One of the limitations of this study is that since it was an in vitro study, the results would have been altered if the study was performed under clinical settings such as effect of sulcus depth. Other limitations were that the accuracy of the master casts obtained was evaluated in relation to a reference model with implants placed in ideal parallel positions at similar gingival levels, unlike in clinical situations which make it impossible for clinicians to place implants with absolute parallelism exhibiting varying angulations and dissimilar gingival levels. Further studies are required to assess the effect of implant angulation on the distortion of impression materials. In the present study, stock trays produced similar results as obtained with custom trays that can be attributed to limited sample size. With usage of stock trays, the impression material used was inculcating additional cost. However, its clinical significance needs to be further addressed. Therefore, to validate this study, a long-term clinical trial should be performed based on preliminary data obtained from this study.

## 5. Conclusions

Within the limitation of present in vitro study, the following conclusions can be drawn.

1. The casts obtained from impressions made with polyether impression material proved to be more accurate statistically than casts obtained from vinylsiloxanether impression material.
2. Statistically similar results were obtained using stock and custom trays when used with medium viscosity impression materials. Therefore, rigid nonperforated stock trays could be an alternative for custom trays for splinted implant impressions.
3. Stock trays when chosen appropriately could show favourable results comparable to custom trays. Further, use of stock trays will save lot of clinician time, which unduly goes in fabrication of casts and custom trays fabrication. Moreover, it is easy to use in a clinical setup.

## Figures and Tables

**Table 1:** Two-way analysis of variance (*µ*m).

Source	Type III sum of squares	df	Mean square	*F*	Sig.
Corrected model	.554^a^	4	.139	3.212	.024
Intercept	24.513	1	24.513	568.337	.000
Impression material	.467	1	.467	10.834	.002
Impression tray	.087	3	.029	.671	.576
Error	1.510	35	.043		
Total	2.064	39			

^a^ *R*-squared.

**Table 2:** Descriptive mean analysis of test impression materials (*µ*m).

Material	*N*	Mean	SD
PE	20	112	7.74
VSXE	20	148	7.74

**Table 3:** Descriptive mean analysis of test impression materials (*µ*m).

Trays	*N*	Mean	SD
Custom			
Self-cure	10	132	.04701
Light cure	10	118	.03171
Total	20	125	.03965

Stock trays			
Metal	10	140	.04086
Plastic	10	131	.03488
Total	20	135	.03726
